# Current Challenges in the Management of Vitreoretinal Conditions

**DOI:** 10.3390/jcm13041171

**Published:** 2024-02-19

**Authors:** Georgios D. Panos

**Affiliations:** 1 Department of Ophthalmology, Queen’s Medical Centre, Nottingham University Hospitals, Nottingham NG7 2UH, UK; gdpanos@gmail.com; Tel.: +44-115-924-9924; 2 Division of Ophthalmology and Visual Sciences, School of Medicine, University of Nottingham, Nottingham NG7 2UH, UK

## 1. Introduction

In the dynamic realm of ophthalmology, the management of vitreoretinal conditions stands as a testament to both significant progress and ongoing challenges. These conditions, encompassing a diverse range of retinal and vitreous pathologies, are crucial in their impact on vision and in the complexity of their care. From diabetic retinopathy and age-related macular degeneration to retinal detachments and rare inherited disorders, the spectrum of vitreoretinal diseases poses unique challenges to clinicians and researchers alike.

The current state of the field is marked by rapid advancements and evolving practices. The past decade has seen considerable strides in diagnostic imaging, with technologies like optical coherence tomography (OCT) and OCT-angiography providing unprecedented insights into retinal structures [1]. Similarly, the advent of anti-VEGF therapies has revolutionised the treatment of conditions such as diabetic macular oedema and neovascular AMD, offering patients hope for preserved vision that was once inconceivable [2,3,4].

However, despite these advancements, the field continues to grapple with significant challenges. Diagnostically, the subtleties of vitreoretinal conditions often demand a level of precision that remains just beyond the reach of current technologies. The heterogeneous nature of these diseases, coupled with individual variations in patient response to treatment, further complicates the clinical landscape. Clinicians must navigate these complexities while also considering factors such as patient comorbidities, accessibility to care, and treatment adherence.

A critical challenge that has recently emerged is the impact of the COVID-19 pandemic. The pandemic has disrupted routine healthcare, leading to delayed diagnoses and treatments for vitreoretinal conditions [5,6]. Additionally, the potential direct and indirect effects of COVID-19 on the retina and its vasculature are areas of active investigation. The necessity for remote consultations and the limitation of in-person clinical interactions have accelerated the adoption of telemedicine, presenting both opportunities and challenges in the management of these conditions [7].

In terms of treatment, while anti-VEGF therapies have been a game-changer, their long-term efficacy, optimal dosing schedules, and systemic implications are areas of ongoing research [8]. Surgical interventions, though continually improving, still carry inherent risks and limitations, particularly in complex or advanced cases. Furthermore, the management of vitreoretinal conditions in paediatric populations presents an additional layer of complexity, given the differences in disease manifestation and treatment responses when compared to those of adults [9,10].

Moreover, the field is at a crossroads with regard to public health implications. As the global population ages, the prevalence of age-related vitreoretinal conditions is expected to rise, posing significant challenges in terms of healthcare resource allocation and patient management on a broader scale [11,12]. The intersection of systemic diseases such as diabetes with retinal health further underscores the need for an integrated approach to patient care, one that transcends the traditional boundaries of ophthalmology.

While the management of vitreoretinal conditions has advanced considerably, it remains a field defined by its challenges. These challenges span the spectrum from the molecular underpinnings of diseases to the practical realities of clinical care, public health, and the recent impact of the COVID-19 pandemic. As we continue to navigate this complex landscape, our focus must remain on the continued pursuit of innovation, improved patient outcomes, and the expansion of our understanding of these intricate conditions.

## 2. An Overview of Published Articles

Mastropasqua et al. (Contributor 1) conducted a study focusing on the short-term effects of a novel implantable miniature telescope (SING-IMT™) in patients with end-stage age-related macular degeneration (AMD) and cataract. They reported significant improvements in both distance and near visual acuity and manageable intraocular pressure increases, recommending intraoperative mechanical iridectomy for better post-operative management.

Yamakawa et al. (Contributor 2) identified immune mediator profiles in the aqueous humour of eyes with simple diabetic retinopathy (DR). Their research, comparing 15 eyes with simple DR against 22 control eyes, found ten immune mediators in higher concentrations in the DR group, suggesting the importance of monitoring these mediators even in early stages of DR.

Sedova et al. (Contributor 3) evaluated changes in surgical strategies for primary rhegmatogenous retinal detachment (RRD) treatment over eight years. Their retrospective analysis of 812 cases showed improved visual acuity and reattachment rates, with a shift from scleral buckling to primary vitrectomy and an increased preference for daytime surgeries.

Merad et al. (Contributor 4) focused on cystoid macular oedema (CMO) post pars plana vitrectomy (PPV) for primary rhegmatogenous retinal detachment repair. In their study involving 493 eyes, they identified risk factors for developing CMO, including worse presenting visual acuity and specific surgical techniques.

Hsu et al. (Contributor 5) investigated the treatment outcomes and complications of peripheral circumferential retinal detachment (PCD) following successful vitrectomy. They concluded that proper management, including peripheral retinectomy, is vital for preserving visual function in PCD cases.

Cisiecki et al. (Contributor 6) assessed the effectiveness of encircling scleral buckling surgery for severe hypotony caused by proliferative vitreoretinopathy-induced retinal detachment. Their retrospective study of six patients showed improved intraocular pressure and visual acuity post-surgery, suggesting the potential usefulness of this surgical technique in specific cases of severe hypotony.

Our research team (Contributor 7) reported on the significant impact of the COVID-19 pandemic on macular hole surgery. Our study compared pre-pandemic and pandemic-period data, highlighting a marked reduction in surgeries, increased waiting times, and poorer surgical outcomes during the pandemic, underscoring the need for better strategies to continue elective surgeries during such crises.

Raimondi et al. (Contributor 8) conducted a literature review on the FIL SSF (Carlevale) intraocular lens, comparing its outcomes with those for other secondary IOL implants. Their analysis revealed comparable outcomes and lower rates of post-operative complications with the FIL SSF IOL, indicating its effectiveness and safety in cases lacking capsular support.

Confalonieri et al. (Contributor 9) undertook a systematic review examining treatment outcomes for subretinal macular haemorrhage (SRMH) secondary to AMD. Despite various treatment approaches, there is no consensus on the best treatment modality, highlighting the need for further research.

Lastly, Ripa et al. (Contributor 10) performed a systematic review and meta-analysis to evaluate the incidence of sympathetic ophthalmia (SO) following vitreoretinal surgery in the absence of trauma. Their study indicated that VR surgery could be a trigger for SO, emphasising the need for careful patient counselling in VR surgery.

## 3. Conclusions

In conclusion, the articles featured in this Special Issue collectively underscore the multifaceted and evolving landscape of vitreoretinal conditions. The diversity of topics, ranging from advanced surgical techniques, as explored by Mastropasqua et al., to the intricate interplay of immune mediators in diabetic retinopathy investigated by Yamakawa et al., highlights the breadth and complexity of challenges faced in this field. The shift in surgical paradigms towards more effective and less invasive techniques, demonstrated in the works of Sedova et al. and Cisiecki et al., reflects a broader trend towards patient-centred care that maximises outcomes while minimising risks.

The impact of the COVID-19 pandemic, as discussed by our research team, has brought to light the vulnerabilities and resilience of healthcare systems, underscoring the necessity for adaptable and robust strategies to maintain high-quality care even in times of crisis. This is particularly pertinent given the time-sensitive nature of many vitreoretinal interventions, where delays can lead to significantly poorer outcomes.

The collective insights from these studies not only enhance our understanding of the current state of vitreoretinal management but also pave the way for future research and innovation. The need for continued exploration is evident, as seen in the systematic reviews by Confalonieri and Ripa et al., which call for more definitive research to establish optimal treatment protocols for complex conditions like subretinal macular haemorrhage and to better understand rare but serious complications like sympathetic ophthalmia post-surgery.

As we navigate the intricate interplay of clinical practice, research advancements, and healthcare delivery, the articles in this Special Issue offer valuable guidance and inspiration. They remind us of the importance of ongoing research, the need for the continued development of more effective and safer treatment modalities, and the critical role of adapting to new challenges, such as those presented by global health crises.

In summary, this Special Issue not only provides a comprehensive overview of the current challenges in the management of vitreoretinal conditions but also serves as a beacon, guiding us towards a future where improved understanding and innovative approaches can lead to better patient outcomes and a deeper comprehension of these complex conditions.
